# Publisher Correction: Genomic epidemiology of SARS-CoV-2 in the UAE reveals novel virus mutation, patterns of co-infection and tissue specific host immune response

**DOI:** 10.1038/s41598-021-98373-2

**Published:** 2021-09-15

**Authors:** Rong Liu, Pei Wu, Pauline Ogrodzki, Sally Mahmoud, Ke Liang, Pengjuan Liu, Stephen S. Francis, Hanif Khalak, Denghui Liu, Junhua Li, Tao Ma, Fang Chen, Weibin Liu, Xinyu Huang, Wenjun He, Zhaorong Yuan, Nan Qiao, Xin Meng, Budoor Alqarni, Javier Quilez, Vinay Kusuma, Long Lin, Xin Jin, Chongguang Yang, Xavier Anton, Ashish Koshy, Huanming Yang, Xun Xu, Jian Wang, Peng Xiao, Nawal Al Kaabi, Mohammed Saifuddin Fasihuddin, Francis Amirtharaj Selvaraj, Stefan Weber, Farida Ismail Al Hosani, Siyang Liu, Walid Abbas Zaher

**Affiliations:** 1 Group42 Healthcare, Abu Dhabi, United Arab Emirates; 2 BGI-Shenzhen, Shenzhen, 518083 Guangdong China; 3 MGI, BGI-Shenzhen, Shenzhen, 518083 Guangdong China; 4 Department of Neurological Surgery, University of California, San Francisco, USA; 5 Department of Epidemiology and Biostatistics, University of California, San Francisco, USA; 6 Laboratory of Health Intelligence, Huawei Technologies Co., Ltd., Shenzhen, 518100 China; 7 Shenzhen Key Laboratory of Unknown Pathogen Identification, BGI-Shenzhen, Shenzhen, 518083 China; 8 Department of Epidemiology of Microbial Diseases, Yale School of Public Health, New Haven, USA; 9 SEHA, Abu Dhabi Health Services Co., Sheikh Khalifa Medical City, Abu Dhabi, United Arab Emirates; 10 Department of Health, Abu Dhabi, United Arab Emirates; 11 School of Public Health (Shenzhen), Sun Yat-sen University, Shenzhen, 510006 Guangdong China

Correction to: *Scientific Reports* 10.1038/s41598-021-92851-3, published online 07 July 2021

References 4, 16, 18, 19 and 20 were incorrectly included in the original version of this Article, in the Results and Methods section, and have subsequently been removed.

The original Article has been corrected.

